# Preeclampsia in Ghana: A study on the prevalence and risk factors of postpartum readmission in the Ashanti Region

**DOI:** 10.1002/puh2.154

**Published:** 2024-01-24

**Authors:** Enoch Odame Anto, Wina Ivy Ofori Boadu, Lovelace Kwaku Gyamfi, Emmanuel Ekow Korsah, Ezekiel Ansah, Joseph Frimpong, Valentine Christian Kodzo Tsatsu Tamakloe, Augustine Tawiah, Elizabeth Aboagye, Albright Etwi‐Mensah, Agartha Odame Anto, Christian Obirikorang

**Affiliations:** ^1^ Department of Medical Diagnostics Faculty of Allied Health Sciences College of Health Sciences Kwame Nkrumah University of Science and Technology Kumasi Ghana; ^2^ School of Medical and Health Sciences Edith Cowan University Joondalup Perth Australia; ^3^ Centre for Precision Health ECU Strategic Research Centre Edith Cowan University Joondalup Perth Australia; ^4^ Komfo Anokye Teaching Hospital Kumasi Ghana; ^5^ Department of Obstetrics and Gynaecology Ho Teaching Hospital Ho Ghana; ^6^ Department of Molecular Medicine School of Medicine and Dentistry College of Health Science Kwame Nkrumah University of Science and Technology Kumasi Ghana

**Keywords:** hypertension, maternal and child health, postpartum readmission, preeclampsia, pregnancy, prevalence, risk factors

## Abstract

**Background:**

Despite significant improvements in its management, preeclampsia continues to be one of the frequent causes of postpartum readmission. Increased awareness of the risk factors for postpartum readmission is required to reduce its incidence and related complications. This study determined the prevalence and associated risk factors for postpartum readmission among women with preexisting preeclampsia in a Ghanaian population.

**Methods:**

This hospital‐based retrospective study was conducted at the obstetrics and gynaecology units of Komfo Anokye Teaching Hospital in Ghana. We reviewed the medical records including sociodemographic and obstetric characteristics of preeclampsia patients. Statistical analyses were performed using Statistical Package for Social Sciences Version 26.0 and GraphPad Prism version 8.0. *p*‐Value <0.05 was considered statistically significant.

**Results:**

A total of 208 preeclampsia patients were considered in this study. The rate of postpartum readmission for a hypertensive disorder among women with preeclampsia was 46%. After adjusting for multiple confounders in the multivariate logistic model, having a family history of hypertension [aOR = 3.512, 95% CI (1.669–7.394), *p* = 0.0028] was associated with increased odds for postpartum readmission. However, being nulliparous [aOR = 0.321, 95% CI (0.116–0.887), *p* = 0.0028] was independently associated with a reduced odd for postpartum readmission.

**Conclusion:**

The rates of postpartum readmission are significantly high among preeclampsia women. Our findings highlight the importance of continued efforts to improve the health outcomes of both mother and newborn, including heightened monitoring of at‐risk patients in the Ghanaian population.

## Graphical abstract

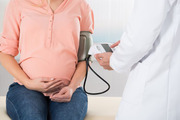

## INTRODUCTION

Globally, hypertensive disorders in pregnancy are a leading cause of adverse foetal, neonatal and maternal outcomes [1, 2]. Preeclampsia, the most frequent hypertensive disorder, complicates 2%–8% of all pregnancies and accounts for about 14% of maternal deaths and 12% of perinatal mortality [3, 4, 5]. Preeclampsia is defined as new‐onset hypertension presenting after 20 weeks of gestation together with at least one of the following characteristic features; such as proteinuria (300 mg/day); and/or maternal organ dysfunction (including renal insufficiency, liver involvement, neurological, or haematological complications); eclampsia (hypertension associated seizures in pregnancy), or uteroplacental dysfunction [6]. The underlying pathophysiology of preeclampsia has been proposed to be a result of an abnormal trophoblastic invasion during placental implantation, leading to placenta ischemia and insufficiency, with subsequent endothelial injury [7, 8, 9].

The incidence of preeclampsia is estimated to be seven times higher in low‐ and middle‐income countries than in developed countries [10]. In Africa, the prevalence of preeclampsia ranges from 1.8% to 16.7% [11, 12]. Early pregnancies, multiparity, older maternal age, maternal anaemia and infections, and low socioeconomic status are all significant risk factors for preeclampsia that are very frequent among African women [13], and this contributes to the high incidence of preeclampsia in this region. In Ghana, the prevalence of preeclampsia is estimated to be between 6.55% and 7.3%, making it one of the major causes of maternal and neonatal deaths [14, 15].

Although most cases of preeclampsia are mild and resolve after delivery, some women may experience severe complications requiring hospital readmission [16]. About 30% of cases of postpartum readmission are attributed to preeclampsia together with other hypertensive disorders in pregnancy [17]. Postpartum readmission represents a poor hypertensive control due to the worsening or persistence of preexisting preeclampsia within 42 days after birth [16, 17, 18] and increases the risk for adverse complications such as seizure, renal failure and pulmonary oedema. Moreover, postpartum readmission notably hinders the usual postpartum recovery process and can result in a significant financial burden [19, 20].

Nevertheless, there is a lack of data on the prevalence and risk factors of postpartum readmission among preeclamptic women in Sub‐Saharan Africa, a setting where the incidence of readmission and maternal deaths is high. In Ghana, knowledge on the rates of postpartum readmission and its risk factors among women with preexisting preeclampsia is non‐existent. Effective prevention programmes for postpartum readmission require increased awareness of factors associated with early postpartum readmission. Hence, this study assessed the prevalence of postpartum readmission and associated risk factors among women with preexisting preeclampsia in a Ghanaian population. This finding will help identify at‐risk patients, thereby developing consistent management strategies and guidelines to reduce postpartum readmission and overall morbidity and mortality rates.

## METHODS

### Study design, setting and population

This hospital‐based retrospective cross‐sectional study was conducted at the obstetrics and gynaecology units at Komfo Anokye Teaching Hospital (KATH). Participants’ data were retrieved from hospital archives and patient's records. KATH is the second major tertiary hospital in Ghana. The facility has over 1200 bed capacity and serves as a referral centre for other hospitals in the middle and northern belts of Ghana. In addition, it receives referrals from neighbouring countries such as Burkina Faso and Ivory Coast.

The study population included women with preexisting preeclampsia before delivery and hospitalization from 2019 to 2021, and participants were selected using convenience sampling. Using a single population formula, this study considered: The prevalence of preeclampsia in a previous study conducted in Ghana (*p* = 0.073). Margin of error (*D* = 0.05), level of significance (*α* = 0.05), *Z α*/2 at 95% CI = 1.96% and 10% contingency rate using the Cochrane formula, *n* = $\frac{Z^{2}P\left(1-P\right)}{D^{2}}$, a sample size of 103 was obtained. To increase statistical power, a total of 208 women with preexisting preeclampsia were included in the study.

Records of women who were included in the study were those who had delivered within the study period, discharged from th hospital after delivery and were diagnosed with preeclampsia during antenatal care prior to delivery hospitalization. We excluded the records of women with twin pregnancies, non‐Ghanaians, women who died before being discharged (as readmission is impossible) as well as women with incomplete important data from delivery admission or readmission. Additionally, readmissions for reasons without or unrelated to hypertension such as infection, headache, venous thromboembolism or cardiac disease were excluded.

### Study variables and clinical data collection

Medical files from the maternal birth register were inspected, and information on age, gender, religion, level of education, occupation and ethnicity were abstracted. Obstetric information, including parity, gravidity, gestational age of childbirth, maternal pre‐pregnancy height and weight, prior preeclampsia in previous pregnancy, prior stillbirth, family history of hypertension, readmission status and maternal medical history, were also obtained.

Preeclampsia was defined based on the revised definition by the International Society for the Study of Hypertension in Pregnancy as a new‐onset of gestational hypertension (140 mmHg systolic/90 mmHg diastolic) developed at or after 20 weeks gestation and with new‐onset of at least one of proteinuria maternal organ dysfunctions (neurological complications, pulmonary oedema, haematological complications, liver involvement or acute kidney injury) and/or uteroplacental dysfunction [6]. Postpartum readmission was defined as admission to the hospital within 42 days post‐discharge from delivery admission/hospitalization for worsening or persistent hypertensive disorder [17, 18].

### Data analysis

The collected data obtained were entered, coded, edited and cleaned in Microsoft Excel 2019. All statistical analyses were performed using the Statistical Package for Social Sciences (SPSS) Version 26.0 and GraphPad Prism version 8.0.1 (GraphPad Software, www.graphpad.com). Categorical data were presented as frequencies and percentages, whereas continuous variables as ± standard deviation for parametric variables and median (Interquartile range) for non‐parametric variables. A bar chart was used to illustrate the prevalence of postpartum readmission among study participants. The Chi‐square test/Fischer's exact test and the univariate followed by multivariate logistic regression analysis were employed to test for associations and the strength thereof between maternal sociodemographic and obstetrics and postpartum readmission. The *p*‐values less than 0.05 were considered statistically significant.

### Ethical considerations

The study was approved by the KATH Institutional Review Board (KATH/IRB/AP/095/22). Confidentially, subjects’ information and anonymity were ensured.

## RESULTS

### Sociodemographic and obstetric characteristics of the study participants

A total of 208 women with preeclampsia were included in this study. About one‐fourth of the study participants were in the age categories of 35–39 (27.9%) and 30–34 (23.6%). Nonetheless, age categories were not associated with readmission status (*p* = 0.712). The majority of the participants attended secondary school (38.0%), were employed in the informal sector (54.3%), were Akans (67.8%) or did not consume alcohol (98.1%). Similarly, educational level, occupation, ethnicity and alcohol consumption were not associated with readmission status (*p* < 0.05) (Table 1).

**TABLE 1:** Sociodemographic and obstetric characteristics of the study participants.

		Readmission status		
Variables	Total (*n* = 208)	No (*n* = 112)	Yes (*n* = 96)	*p‐*Value
**Maternal age (years)**	31.43 ± 6.87	31.33 ± 6.98	31.54 ± 6.781	0.826
**Maternal age group (years)**				0.712
<25	43 (20.7)	24 (21.4)	19 (19.8)	
25–29	38 (18.3)	22 (19.6)	16 (16.7)	
30–34	49 (23.6)	24 (21.4)	25 (26)	
35–39	58 (27.9)	29 (25.9)	29 (30.2)	
≥40	20 (9.6)	13 (11.6)	7 (7.3)	
**Educational level**				0.124
No formal education	31 (14.9)	20 (17.9)	11 (11.5)	
Primary school	48 (23.1)	19 (17)	29 (30.2)	
Secondary school	79 (38.0)	45 (40.2)	34 (35.4)	
Tertiary	50 (24.0)	28 (25)	22 (22.9)	
**Occupation**				0.551
Unemployed	44 (21.2)	25 (22.3)	19 (19.8)	
Informal	113 (54.3)	57 (50.9)	56 (58.3)	
Formal	51 (24.5)	30 (26.8)	21 (21.9)	
**Ethnicity**				0.636
Ewe	7 (3.4)	4 (3.6)	3 (3.1)	
Akan	141 (67.8)	79 (70.5)	62 (64.6)	
Mole‐Dagbani	34 (16.3)	18 (16.1)	16 (16.7)	
Ga‐Adangbe	26 (12.5)	11 (9.8)	15 (15.6)	
**Alcohol consumption**				0.629
No	204 (98.1)	110 (98.2)	94 (97.9)	
**Maternal BMI (kg/m^2^)**				0.6930
Underweight	4 (1.9)	3 (2.7)	1 (1)	
Normal weight	95 (45.7)	51 (45.5)	44 (45.8)	
Overweight	79 (38)	44 (39.3)	35 (36.5)	
Obesity	30 (14.4)	14 (12.5)	16 (16.7)	
**Gravidity status**				**0.0020**
Primigravida	10 (4.8)	10 (8.9)	0 (0)	
Multigravida	136 (65.4)	76 (67.9)	60 (62.5)	
Grand multigravida	62 (29.8)	26 (23.2)	36 (37.5)	
**Parity groups**				**0.0004**
Nulliparous	37 (17.8)	29 (25.9)	8 (8.3)	
Primiparous	65 (31.3)	33 (29.5)	32 (33.3)	
Multiparous	106 (51)	50 (44.6)	56 (58.3)	
**PPE**				**0.0130**
No	39 (18.8)	28 (25)	11 (11.5)	
Yes	169 (81.3)	84 (75)	85 (88.5)	
**Prior stillbirth**				0.6590
No	140 (67.3)	77 (68.8)	63 (65.6)	
Yes	68 (32.7)	35 (31.3)	33 (34.4)	
**ANC visit**				
More than four times	38 (18.3)	22 (19.6)	16 (16.7)	0.5950
Less than four times	170 (81.7)	90 (43.3)	80 (83.3)	
**Family history of HTN**				**0.0001**
No	46 (22.1)	35 (31.3)	11 (11.5)	
Yes	162 (77.9)	77 (68.8)	85 (88.5)	
**Maternal medical history**				0.2590
None	49 (23.6)	29 (25.9)	20 (20.8)	
Chronic hypertension	73 (35.1)	35 (31.3)	38 (39.6)	
Diabetes	16 (7.7)	9 (8)	7 (7.3)	
Kidney diseases	14 (6.7)	7 (6.3)	7 (7.3)	
STIs	13 (6.2)	8 (7.2)	5 (5.2)	
Others	43 (20.7)	24 (21.4)	19 (19.8)	
**Anaemia status**				0.2510
Anaemic	205 (98.6)	109 (97.3)	96 (100)	
Non‐anaemic	30 (1.4)	30 (2.7)	0 (0)	
**Gestational age**				0.5580
Preterm	167 (80.30)	90 (80.40)	77 (80.20)	
Term	41 (19.70)	22 (19.60)	19 (19.80)	

*Note*: Data is presented as mean ± SD; independent sample *t*‐test, frequency (%); chi square/Fisher's exact test, *p*‐value <0.05 was considered significant for postpartum readmission.

Abbreviations: n, number; SD, standard deviation.

Bold *p*‐values indicate statistical significance.

Majority of the participants had normal weight (45.7%) followed by overweight (38.0%), and a few were underweight (1.9%). Majority of the participants were multigravida (65.4%), were multiparous (51.0%), had prior preeclampsia in their previous pregnancy (81.3%) and had prior stillbirth (67.3%) or a family history of hypertension (77.9%). Most of the participants were anaemic (98.6%) or had a preterm birth (80.30%). Gravidity status, parity groups, family history of hypertension and prior preeclampsia were associated with readmission status among the study participants (*p* < 0.05) as shown in Table 1.

### Prevalence of postpartum readmission amongst the preeclamptic women

**FIGURE 1:**
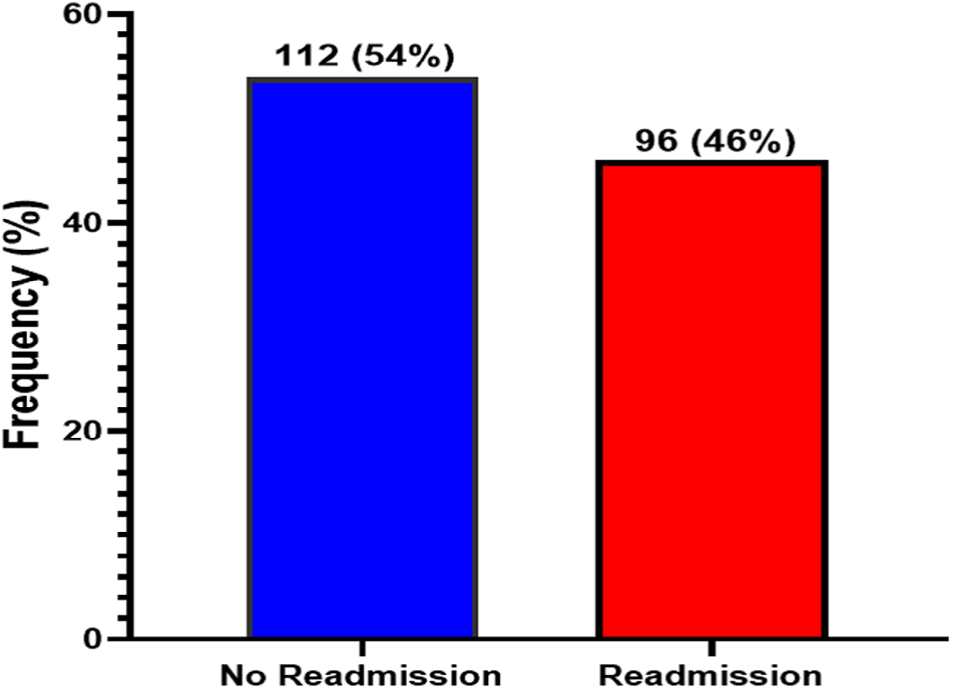
Prevalence of postpartum readmission amongst the preeclamptic women.

Figure 1 shows the prevalence of postpartum readmission among women with antepartum preeclampsia. Of the 208 preeclamptic women, 96 (46%) were readmitted for a hypertensive disorder, whereas 112 (54%) of the participants were not readmitted.

### Obstetric characteristics associated with postpartum readmission among preeclamptic women

In the univariate logistic regression model, being nulliparous, having prior preeclampsia and having a family history of hypertension were associated with postpartum readmission.

After adjusting for multiple confounders in the multivariate logistic model, being nulliparous [aOR = 0.321, 95% CI (0.116–0.887), *p* = 0.0028] was independently associated with a decreasing odd for postpartum readmission. However, having a family history of hypertension [aOR = 3.512, 95% CI (1.669–7.394), *p* = 0.0028] was associated with increasing odds for postpartum readmission (Table 2).

**TABLE 2:** Obstetric characteristics associated with postpartum readmission among preeclamptic women.

Variable	cOR (95% CI)	*p‐*Value	aOR (95% CI)	*p*‐Value
**Parity**				
Primiparous	Ref [1]		–	–
Nulliparous	0.246 (0.103–0.588)	**0.002**	0.321 (0.116–0.887)	**0.028**
Multiparous	0.866 (0.467–1.606)	0.648	–	
**Prior preeclampsia**				
No	Ref [1]		–	–
Yes	2.576 (1.205–5.507)	**0.015**	0.966 (0.321–2.909)	0.951
**Family history of hypertension**				
No	Ref [1]		–	–
Yes	3.512 (1.669–7.394)	**0.001**	3.041 (1.229–7.521)	**0.016**

*Note*: Univariate and multivariate logistic regression analysis performed to obtain odd ratios. *p*‐Value of <0.05 was considered statistically significant.

Abbreviations: aOR, adjusted odd ratio; CI, confidence interval; cOR, crude odd ratio.

Bold *p*‐values indicate statistical significance.

## DISCUSSION

The rates of postpartum readmission are a significant measure of the standard or quality of medical care received during pregnancy and after birth. Despite major improvements in its management, preeclampsia remains one of the leading causes of readmission following delivery discharge. However, data on incidence and risk characterization of postpartum readmission is lacking or non‐existent in many Sub‐Saharan African countries. This study characterized the prevalence and risk factors for postpartum readmission for hypertensive disorder among a cohort of women in Ghana with preexisting preeclampsia.

In this study, the prevalence of postpartum readmission among preeclampsia women was found to be 46%. This rate of postpartum readmission is very alarming as it is considerably higher than reports from previous studies in the United States of America, which showed a 3.6%–5.7% incidence of postpartum readmission among preeclampsia women [16, 17, 21]. This high incidence of postpartum readmission in Ghana could be due to the comparatively poor healthcare system [22, 23]. Women in developed countries generally have access to comprehensive prenatal care, which includes regular check‐ups and testing to identify and manage any potential complications, as well as adequate monitoring and support during the postnatal period [24]. These appropriate practices are not religiously observed in Ghana [25]. This suggests that there is more room for improvement in the care provided to women with preeclampsia as well as a transition care plan after delivery. This observed disparity in postpartum readmission rates may also be attributed to the smaller sample size and ethnicity differences.

Although much research has been done on antepartum and intrapartum hypertensive diseases, there is a lack of data on the factors that increase the risk of persistent hypertensive disease during the postpartum period and its associated readmission. It is crucial to identify and treat persistent hypertensive disease early after childbirth; otherwise, it could lead to disease progression, which in turn increases the risk of stroke and seizures [19]. In this study, we found that preeclampsia women with a family history of hypertension are likely to be readmitted after delivery discharge. The association between family history of hypertension and the incidence of preeclampsia has been reported by previous studies [26, 27, 28]. Preeclampsia persists after delivery discharge in women with a family history of hypertension [29]. Our findings agree with this previous study and go further to show that such patients are likely to be readmitted. These findings suggest that healthcare providers should take a woman's family history of hypertension into account when assessing her risk for postpartum readmission. Close monitoring, early intervention and medication management, if necessary, are required to prevent adverse complications and reduce the likelihood of readmission in these high‐risk groups.

In this study, we found that nulliparous women were at a reduced risk for readmission compared to primiparous women after adjusting for possible confounders in the multivariate analysis. This finding is quite unusual because preeclampsia and its complications are often seen as a disease of the first pregnancy [30, 31]. One study reported no significant association between parity groups and readmission [21]. Another study showed no association between the persistence of preeclampsia after delivery and parity groups [29]. These conflicting reports may be due to the small sample size of this study or external factors such as the quality of prenatal care received and dietary and lifestyle practices, which were not assessed in this study. Future prospective studies, taking into consideration all these variables, are required to validate this finding.

This study has limitations. First, we could not determine the quality of care provided to the women, specifically in terms of whether they received sufficient education during discharge about managing their health and monitoring their symptoms regularly. Second, is the absence of important variables related to the post‐discharge period that could affect the likelihood of postpartum readmission, including continuous blood pressure readings over time and measures of the effectiveness of hypertension management. Lastly, the sample size was small which could have introduced a bias in this study.

## CONCLUSION

The rates of postpartum readmission are significantly high among preeclampsia women. Our findings highlight the need for continued efforts to improve maternal and neonatal health outcomes in low‐ and middle‐income countries. Enhancing patient education, improving contact time with health providers and monitoring at‐risk patients, including those with a family history of hypertension, before and after discharge can prevent readmissions and reduce maternal morbidity and mortality. This requires increased provider–patient communication and contact time.

## AUTHOR CONTRIBUTIONS

*Conceptualization; methodology; project administration; supervision; writing—review and editing*: Enoch Odame Anto. *Methodology; data curation; supervision; writing—review and editing*: Wina Ivy Ofori Boadu. *Conceptualization; methodology; data curation; writing—review and editing*: Lovelace Kwaku Gyamfi. *Formal analysis; writing—original draft; writing—review and editing*: Emmanuel Ekow Korsah and Ezekiel Ansah. *Methodology; formal analysis; writing—review and editing*: Joseph Frimpong. *Methodology; data curation; writing—review and editing*: Valentine Christian Kodzo Tsatsu Tamakloe and Augustine Tawiah. *Conceptualization; writing—review and editing*: Elizabeth Aboagye. *Data curation; writing—original draft; writing—review and editing*: Albright Etwi‐Mensah. *Data curation; resources; writing—review and editing*: Agartha Odame Anto. *Conceptualization; project administration; writing—review and editing*: Christian Obirikorang.

## CONFLICT OF INTEREST STATEMENT

None declared.

## FUNDING INFORMATION

This study did not receive any external funding.

## ETHICS STATEMENT

The study was approved by the Komfo Anokye Teaching Hospital Institutional Review Board (KATH/IRB/AP/095/22). Confidentiality, subjects’ information and anonymity were ensured.

## Data Availability

All data generated and analysed during this study are included in the manuscript and can be requested from the corresponding author.
